# Sodium ferulate lowers portal pressure in rats with secondary biliary cirrhosis through the RhoA/Rho-kinase signaling pathway: A preliminary study

**DOI:** 10.3892/ijmm.2014.1905

**Published:** 2014-08-19

**Authors:** LAI WEI, JUAN YANG, MIN WANG, SHENG-NAN XU, HUA-MIN LIANG, QI ZHOU

**Affiliations:** 1 Institute of Organ Transplantation, Tongji Hospital, Tongji Medical College, Huazhong University of Science and Technology, Wuhan, Hubei, P.R. China; 2 Department of Digestive Diseases, Tongji Hospital, Tongji Medical College, Huazhong University of Science and Technology, Wuhan, Hubei, P.R. China; 3 Department of Digestive Diseases, Chengdu First People’s Hospital, Chengdu, Sichuan, P.R. China; 4 Department of Emergency Medicine, Tongji Hospital, Tongji Medical College, Huazhong University of Science and Technology, Wuhan, Hubei, P.R. China; 5 Department of Physiology, Tongji Medical College, Huazhong University of Science and Technology, Wuhan, Hubei, P.R. China

**Keywords:** portal hypertension, sodium ferulate, cirrhosis, bile duct ligation, RhoA, Rho-kinase, methoxamine, endothelial nitric oxide synthase, fibrosis

## Abstract

Cirrhotic rats show higher expression levels of hepatic RhoA and Rho-kinase than normal healthy rats, and the activation of this signaling pathway leads to portal hypertension. Sodium ferulate (SF) has been shown to decrease the production of geranylgeranyl pyrophosphate (GGPP), a substance essential for RhoA activation. In the present study, to investigate the effects of SF on fibrosis, portal hypertension and the RhoA/Rho-kinase pathway, hepatic cirrhosis was induced in rats by bile duct ligation. Liver function and fibrogenesis-related biochemical parameters, the hepatic hydroxyproline content, the pathological characteristics of the liver sections and the levels of hepatic α-smooth muscle actin (α-SMA; by immunohistochemistry) were analyzed to assess effects of SF on hepatic fibrosis. In addition, hepatic RhoA, Rho-kinase and endothelial nitric oxide synthase (eNOS) expression was examined by immunohistochemistry. Apoptosis in the SF-treated and SF + GGPP-treated rat primary hepatic stellate cells (HSCs) and a human stellate cell line (LX-2) was examined by flow cytometry. Intrahepatic resistance and responsiveness to the α1-adrenoceptor agonist, methoxamine, were investigated by *in situ* liver perfusion. Treatment with SF did not affect fibrosis-related biochemical parameters or the hydroxyproline content; however, SF reduced the histological evidence of fibrosis and hepatocyte damage. The SF-treated rats had a significantly lower expression of α-SMA and Rho-kinase, as well as an increased hepatic eNOS content; however, SF did not affect RhoA expression. The SF-treated HSCs had a significantly increased apoptotic rate compared to the untreated rats. Following the addition of GGPP, the rate apoptotic rate decreased. SF reduced basal intrahepatic resistance and the responsiveness of hepatic vascular smooth muscle to methoxamine. Therefore, our data demonstrate that SF reduces fibrogenesis, decreases portal pressure in cirrhotic rats and inhibits the activation of the RhoA/Rho-kinase signaling pathway.

## Introduction

The two major features of hepatic cirrhosis are portal hypertension and fibrosis. Portal hypertension is due partly to a fibrosis-induced narrowing of hepatic venules and partly to an increased responsiveness of these venules to vasoconstricting substances (1,2). Currently, the only medications available for the treatment of portal hypertension are non-selective β blockers or vasodilators, such as nitrates (3). Non-selective β blockers decrease blood flow into the portal system through splanchnic vasoconstriction, but do not delay the development of portal hypertension (4,5). Vasodilators can cause arterial hypotension as they exert dilating effects on systemic, as well as portal circulation (4). The current therapeutic approaches do not address the underlying issues, as well as the fibrosis and hypersensitivity to contractile agents.

The RhoA/Rho-kinase pathway in hepatic stellate cells (HSCs) is a potential novel therapeutic target. The number of activated HSCs is increased in cirrhosis (5–8); HSCs produce the increased extracellular matrix responsible for fibrosis (1) and regulate hepatic vascular resistance (9) and sinusoidal tone (10–14). Activated HSCs express high levels of Rho-kinase, and two downstream effects of the RhoA/Rho-kinase pathway are the increase in extracellular matrix formation (1) and vascular hyperreactivity.

The RhoA/Rho-kinase pathway is activated by the binding of a vasoconstrictor to membrane-bound RhoA, a small GTPase protein on the cell surface. However, before binding to a vasoconstrictor can take place, RhoA must be attached to geranylgeranyl pyrophosphate (GGPP), a by-product of cholesterol synthesis, in order to ‘lipidize’ it so that it can be inserted into the cell membrane (15–17). Therefore a drug that blocks cholesterol synthesis at a site upstream from GGPP formation may have the potential to block the RhoA/Rho-kinase pathway and thus attenuate portal hypertension.

Sodium ferulate (SF) is such a drug. It decreases cholesterol synthesis by inhibiting mevalonate 5-pyrophosphate dehydrogenase, an action that prevents the conversion of mevalonite to GGPP and the subsequent activation of the RhoA/Rho-kinase pathway (18).

Based on these data, we hypothesized that SF may decrease fibrosis and portal pressure by inhibiting the RhoA/Rho-kinase pathway. In this study, we administered SF to rats in a bile duct ligation (BDL) model of portal hypertension and examined its effects by measuring indicators of liver function, serum and tissue indicators of fibrosis, immunohistological evidence of RhoA, Rho-kinase and endothelial nitric oxide synthase (eNOS) abundance, as well as responsiveness to the α-adrenergic agonist, methoxamine by *in situ* liver perfusion. In addition, the effects of SF on the apoptosis of *in vitro* cultured rat HSCs and a human hepatic stellate cell line were examined.

## Materials and methods

### Animals and animal model of portal hypertension

Male Wistar rats (180–200 g; n=73) were purchased from the Center for Disease Control of Hubei province, and raised in the Laboratory Animal Centre of Tongji Medical College, Wuhan, China. The study was approved by the Ethics Committee of Tongji Medical College. To induce portal hypertension, the animals were anesthetized intraperitoneally with chloral hydrate, a median laparotomy was performed, the common bile duct was ligated twice and cut between the ligatures, and the abdomen was sutured. The rats were randomly divided into 3 groups. The first group [BDL + normal saline (NS) group, n=28] was subjected to BDL and administered an NS injection via the tail vein for 1 week during the 4th week after ligation. The second group (BDL + SF group, n=21), was subjected to BDL, and a middle dosage of SF (50 mg/kg/day) was injected each day for 1 week during the 4th week after surgery. The third group was the control group [sham-operated (SHAM) + NS group, n=24] which was subjected to a laparotomy without ligation, and an NS injection was administered for 1 week during the 4th week after surgery. At the end of the 4th week, the rats were anesthetized for liver perfusion experiments (described in a later section), or sacrificed after blood collection and the livers and spleens were sampled.

### Serum biochemical parameters and sample preparation

Blood collected from the vena cava was centrifuged for 5 min at 12,000 rpm at 4°C and the serum was then sent to the Clinical Laboratory of Wuhan Tongji Hospital for the measurement of the following substances: alanine aminotransferase (ALT), aspartate aminotransferase (AST), albumin, total bilirubin (TBIL), direct bilirubin (DBIL), γ-glutamyl transferase (GGT); and the fibrogenesis-related compounds hyaluronic acid (HA), laminin (LN), collagen type IV (IV-C), and procollagen type III peptide (PCIII).

The livers and spleens were weighed, and 3–4 liver fragments (0.5×0.5×0.5 cm^3^) were fixed in formalin. The remaining fragments were placed into tubes and quick-frozen in liquid nitrogen. The tissues were stored at −80°C for future analyses.

### Hepatic hydroxyproline content

The hepatic hydroxyproline content was measured using an assay kit (Nanjing Jiancheng Bioengineering Institute, Nanjing, China), according to the manufacturer’s instructions. Briefly, 80–100 mg liver tissue fragments were homogenized, precipitated using trichloroacetic acid and hydrolyzed for 24 h at 110°C in 6 N HCl solution. After hydrolysis was completed, the samples were neutralized with 10 N NaOH, oxidized with chloramine-T, and incubated in Ehrlich’s perchloric acid solution at 65°C for 20 min. The hydroxyproline content was determined photometrically by measuring the absorbance at 560 nm.

### Pathological analysis

Liver tissue, following formalin fixation, was embedded in paraffin and cut into slices (4 μm in thickness). The sections underwent hematoxylin and eosin (H&E) staining, after which the pathological characteristics of the 3 groups were observed under an optical microscope (Olympus, Tokyo, Japan). For ultra microstructure observation by electron microscopy, the rats were anesthetized intraperitoneally with chloral hydrate and the livers were rapidly and gently removed. The liver tissues were then cut into 2×2×3 mm^3^ sections and fixed in 2.5% glutaraldehyde buffer for 5–10 min. Following fixation, the liver became harder, and was then cut into 1 mm^3^ sections or into tissue strips (cross-sectional area, 1 mm^2^; length, 5 mm). After being fixed in 1% osmium tetroxide for 1 h and washed twice with 0.1 M phosphate-buffered saline (PBS) (20 min for each wash), the tissues were treated sequentially in 50% ethanol, 70% ethanol, 90% ethanol, 90% ethanol-acetone, 90% acetone, and then twice in 100% acetone (5 min in each solution). They were then embedded in an epoxy-acetone solution (epoxy:acetone, 1:1) for 2 h, followed by embedding in epoxy for 2 h. After being heated at 80°C for 10 h, the tissues were cut into ultra-thin sections and stained with uranyl acetate and lead citrate (10 min for each). The ultra-structure of the liver was observed under an electron microscope (FEI Tecnai G2 12; FEI Tecnai, Eindhoven, The Netherlands), and images were captured and stored for further analysis.

### Immunohistochemistry

Paraffin-embedded liver tissue was cut into 4 μm-thick slices for staining. Rho-kinase, eNOS and α-smooth muscle actin (α-SMA) (a marker for fibrosis) were stained with a streptavidin peroxidase 3 kit, and RhoA was stained with a Histostain-Plus kit (both kits from Zymed Laboratories, Inc., San Francisco, CA, USA). A diaminobenzidine (DAB) kit (Wuhan Boster Bio-Engineering Ltd., Wuhan, China) was used for color development. The primary antibodies and dilutions used were the following: α-SMA (Wuhan Boster Bio-Engineering Ltd.), 1:100 dilution; Rho-kinase (Abcam, Cambridge, UK), 1:100 dilution; eNOS (Wuhan Boster Bio-Engineering Ltd.), 1:50 dilution; RhoA (Abcam), 1:100 dilution.

For α-SMA, Rho-kinase and eNOS staining, the sections were first deparaffinized in xylene and rehydrated in a graded series of ethanol. Quenching of endogenous peroxidase activity was performed for all specimens in 0.1% hydrogen peroxide diluted in methanol (H_2_O for laminin-5 γ2 chain immunohistochemistry) and non-specific binding was blocked by incubating specimens in 20% fetal calf serum diluted in PBS. Diluted primary antibodies were added to the sections and incubated overnight at 4°C. All specimens were then overlaid with the suitable secondary antibody followed by assay. DAB was used for color reaction, hematoxylin was used for counterstaining. All steps were followed by washes with PBS. Negative controls for all immunostainings were obtained by substituting PBS for the primary antibody. A DAB kit (Wuhan Boster Bio-Engineering Ltd.) was used to visualize positive immunoreaction. In some experiments, nuclei were counterstained with 4′,6-diamidino-2-phenylindole (DAPI; Wuhan Boster Bio-Engineering Ltd.).

For RhoA staining, briefly, endogenous peroxidase activity was blocked by incubation with 3% H_2_O_2_ for 15 min. Antigen retrieval was performed using a microwave. Diluted primary antibodies were added to the sections following by overnight incubation at 4°C. The sections were then incubated in secondary antibody and visualized using the DAB kit. In the control group, the sections were treated as described above but PBS was added instead of the primary antibody.

The sections were visualized under an optical microscope (Olympus). Five fields of vision per section at ×200 magnification were taken blindly. Positive immunostaining was quantified as integrated optical density (IOD) using the Image-Pro Plus analysis system 6.0 (19–21).

### Cell isolation, cultivation and identification

HSC isolation was performed as previously described (22–24). Briefly, male Wistar rats with biliary cirrhosis (4 weeks after surgery; weighing 350–450 g; n=49) were used. Heparin (1,000 units) was injected via the vena cava following anesthesia and laparotomy. A balanced salt solution containing Na^+^ and K^+^ was then injected through the isolated portal vein, and the vena cava was immediately severed. The liver was then perfused with solution containing 0.05 g/100 ml collagenase (Gibco-Invitrogen, Carlsbad, CA, USA) and 0.03 g/100 ml pronase (Roche, Basel, Switzerland). Following perfusion, the liver was cut into sections and incubated with shaking in digestive solution containing DNase (Sigma, St. Louis, MO, USA) at 37°C, after which Dulbecco’s modified Eagle’s medium (DMEM; Gibco-Invitrogen) containing 10% fetal bovine serum (FBS) (Thermo Scientific HyClone, Beijing, China) was used to terminate the reaction. The homogenate was filtered with nylon gauze and centrifuged twice at 50 × g for 4 min. The supernatant was then centrifuged at 500 × g for 7 min. The precipitate was resuspended and the HSCs were isolated by discontinuous Percoll gradient centrifugation at 1,400 × g for 17 min, as previously described (24,25). The cell cluster was collected and resuspended, then centrifuged at 500 × g for 8 min. The cells were then cultured in DMEM containing 15% FBS, 100 U/ml penicillin and 100 U/ml streptomycin under a humidified atmosphere containing 5% CO_2_ and 95% air at 37°C. The medium was replaced after 48 h, then once every other day. Two weeks later, HSC activation was identified by immunofluorescence using primary antibodies to desmin (Wuhan Boster Bio-Engineering Ltd.) and α-SMA. Cytoimmunofluorescence revealed that the proportion of activated HSCs in the cells following culture for 2 weeks was >99%.

### Assessment of apoptosis by flow cytometry

SF powder was pre-dissolved in dimethyl sulfoxide (DMSO) (Sigma), then dissolved in DMEM medium and stored at 4°C away from light. The activated HSCs were divided into the following 3 groups: i) the ‘SF’ group, in which the HSCs were cultured in medium containing SF (40, 120, 360 μg/ml) for 48 h; ii) the‘SF + GGPP’ group, in which the HSCs were treated with 10 μmol/l GGPP and various concentrations of SF for 48 h; iii) the control group, in which the HSCs were cultured in medium alone. For these incubations, medium contained only 2% FBS to support growth. Due to the low concentration of FBS, non-apoptotic cells were in the quiescent phase, instead of a growth phase. The cells that died were identified by flow cytometry, and the proportion of dead cells was <5%. An Annexin V-FITC apoptosis detection kit (KeyGen Biotech, Nanjing, China) was used to detect apoptosis according to the manufacturer’s instructions. Apoptotic cells were detected by flow cytometry.

A human hepatic stellate cell line (LX-2) was purchased from Xiangya Central Experiment Laboratory of Xiangya Medical College, Hunan, China. LX-2 was cultured and cell apoptosis was detected as described above.

### In situ liver perfusion

#### Perfusion system

BL-420E Bio-experimental system software (Chengdu Aimeng Technology Ltd., Chengdu, China) was used to record perfusion pressure in the rat livers. In brief, Krebs-Henseleit bicarbonate buffer [previously described (26)] containing heparin (2 U/ml) was kept in a thermostatic water bath at 37°C and saturated with 95% oxygen and 5% carbon dioxide, as previously described (27,28). A BT100-2J Peristaltic Pump (Lange Peristaltic Pump Inc., Baoding, China) was linked with the T-branch pipe of the BL-420E Bio-experimental system. After making certain that no air was in the working system, the pipe of the peristaltic pump was placed into the perfusate and the basal pressure was set to zero.

#### Rat preparation

Three groups of rats were used for this experiment: the BDL + NS, BDL + SF and the SHAM + NS group. Each group comprised 10 rats. The rats were fasted overnight, but water was supplied as usual. Following anesthetization, a median laparotomy was performed. A PE-50 catheter was introduced into the portal vein and secured. This size catheter was selected as it can be smoothly inserted into the portal vein, but has a large enough diameter so that it has no influence on the resistance of the circulation system. The peristaltic pump was then turned on and the vena cava immediately severed to allow the perfusate to escape. The thoracic cavity was exposed and another catheter was inserted into the right atrium and advanced into the superior vena cava. The basal perfusion pressure was recorded after continuous perfusion for 20 min at a constant flow rate (30 ml/min), as previously described (2). The basal hepatic resistance was calculated according to the following formula: ‘pressure = flow × resistance’, as prevoiusly described (29,30).

When setting up the perfusion process, the following conditions were adhered to in order to ensure the viability and stability of the process: accurate and quick intubation, a sufficiently-oxygenated perfusate with stable pH (7.4±0.1) and a constant temperature of 37°C and no air or impurities in the perfused channel.

### Effects of methoxamine hydrochloride on portal perfusion pressure

After a period of continuous perfusion and allowing the system to stabilize, increasing concentrations (0.1, 1, 10 and 100 μM) of the α1-adrenoreceptor agonist, methoxamine hydrochloride, were added to the perfusate. Each concentration was sustained for 3 min, and the pressure recorded. Changes in intrahepatic resistance were calculated, and differences between groups as regards the effects of methoxamine hydrochloride on intrahepatic resistance were indicated by cumulative concentration-response curves.

### Statistical analysis

Continuous variables are presented as the means ± standard deviation. Comparisons between the 3 experimental groups (SHAM + NS, BDL + NS and BDL + SF groups) were performed by one-way analysis of variance (ANOVA). When a significant difference between groups was observed, multiple comparisons were performed using the Bonferroni procedure with type I error adjustment. The association between the methoxamine concentration and portal perfusion pressure (or intrahepatic resistance) was assessed using the Pearson correlation coefficient. Statistical analyses were performed using SAS software version 9.2 (SAS Institute Inc., Cary, NC, USA). A two-sided P-value <0.05 was considered to indicate a statistically significant difference.

## Results

### General characteristics and serum biochemical parameters

Table I shows the general characteristics and serum biochemistry of the 3 groups. Animals with biliary cirrhosis had a lower body weight, higher liver and spleen weights, lower levels of albumin and higher levels of other indicators of liver damage and fibrosis than the sham-operated rats (all P<0.05). The addition of SF caused no normalization of any of these parameters.

The mean body weight of the BDL + NS and BDL + SF groups was significantly lower and the liver and spleen weights were significantly higher than those of the SHAM + NS group (all P≤0.001). The BDL + NS and BDL + SF groups had significantly higher ALT, AST, TBIL, DBIL and γ-GT concentrations than the SHAM + NS group (all P<0.001). However, the albumin level in the BDL + NS and BDL + SF groups was significantly lower than that of the SHAM + NS group (both P<0.001). The BDL + NS and BDL + SF groups had significantly higher HA, LN, IV-C and PCIII than the SHAM + NS group (all P<0.001).

### Hepatic hydroxyproline content

**Figure 1:**
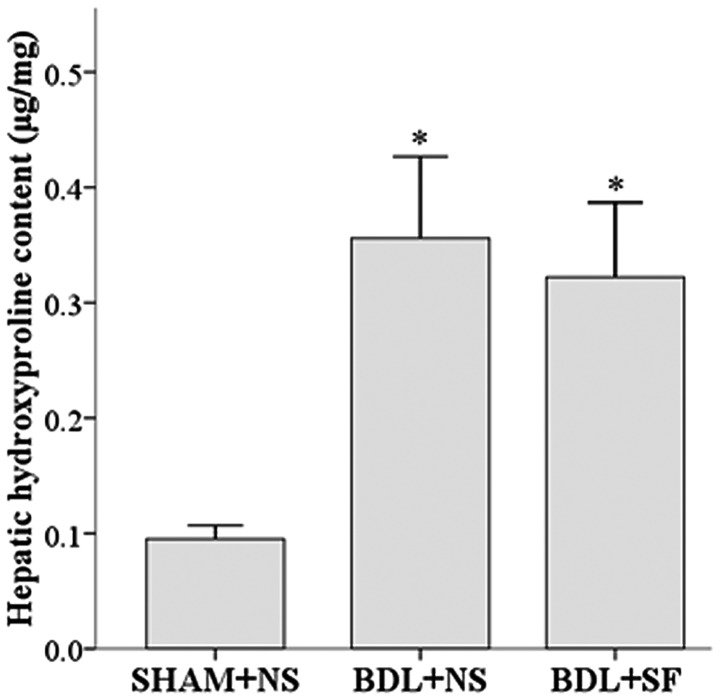
Hepatic hydroxyproline content in liver tissue from the different groups. Hepatic hydroxyproline content in liver tissue from sham-operated + normal saline (SHAM + NS), bile duct ligation + normal saline (BDL + NS) and bile duct ligation + sodium ferulate (BDL + SF) groups. The administration of SF to animals subjected to BDKL had no effect on the hepatic hydroxyproline content. Data are expressed as the means ± standard deviation (SD) (n=8 per group). ^*^P<0.05 vs. SHAM + NS group.

The addition of SF had no effect on the high hepatic hydroxyproline content of the rats with hepatic cirrhosis. The hepatic hydroxyproline content of both the BDL + NS and the BDL + SF groups was significantly higher than that of the SHAM + NS group (both P<0.001) (Fig. 1).

### Pathological analysis

**Figure 2:**
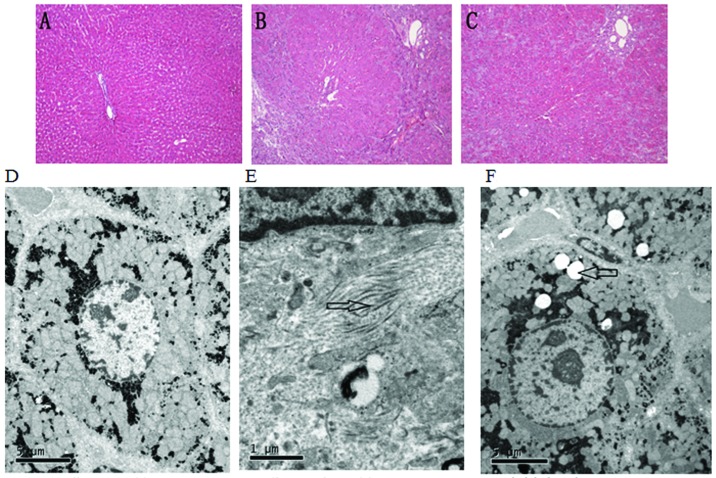
Hepatic hematoxylin and eosin (H&E) staining and ultra microstructure of each group. (A-C) Hepatic H&E staining and (D-F) ultra microstructure of each group. (A and D) Sham-operated + normal saline (SHAM + NS) group; (B and E) bile duct ligation + normal saline (BDL + NS) group; (C and F) bile duct ligation + sodium ferulate (BDL + SF) group. (A-C) Magnification, ×100. (D) Rats in the SHAM + NS group had generally normal hepatocytes; (E) Rats in the BDL + NS group fibrous deposition was observed; (F) in the rats in the BDL + SF group, less fibrous deposition was observed and lipid droplets and mild hepatocyte steatosis were observed.

The histological observations of H&E-stained sections between the different groups were as follows: the sham-operated rats had a normal structure of hepatic lobules and sinusoids, as well as ordered hepatic cords (Fig. 2A). In the cirrhotic rats, hepatic lobules with normal structure were absent, and the proliferation of fibrous tissue, pseudolobule formation and a diffuse distribution of lymphocytes were observed (Fig. 2B). Compared to the untreated cirrhotic rats, the livers of SF-treated rats appeared to have a decreased degree of inflammation, fibrosis and necrosis (Fig. 2C).

Observations using a transmission electron microscope indicated that the hepatocytes in the sham-operated group had abundant organelles, an intact cellular morphology and no fibrous deposition in the perisinusoidal area (Fig. 2D). In the untreated rats subjected to BDL, the organelles were destroyed, the hepatocytes were severely damaged and a large number of collagen fibrils was deposited in the perisinusoidal space (Fig. 2E). Compared to the untreated cirrhotic rats, the SF-treated rats appeared to have less hepatocyte necrosis and fibrous deposition; lipid droplets were also found in the hepatocytoplasm (Fig. 2F).

### Immunohistochemistry for hepatic expression of α-SMA, RhoA, Rho-kinase and eNOS

**Figure 3:**
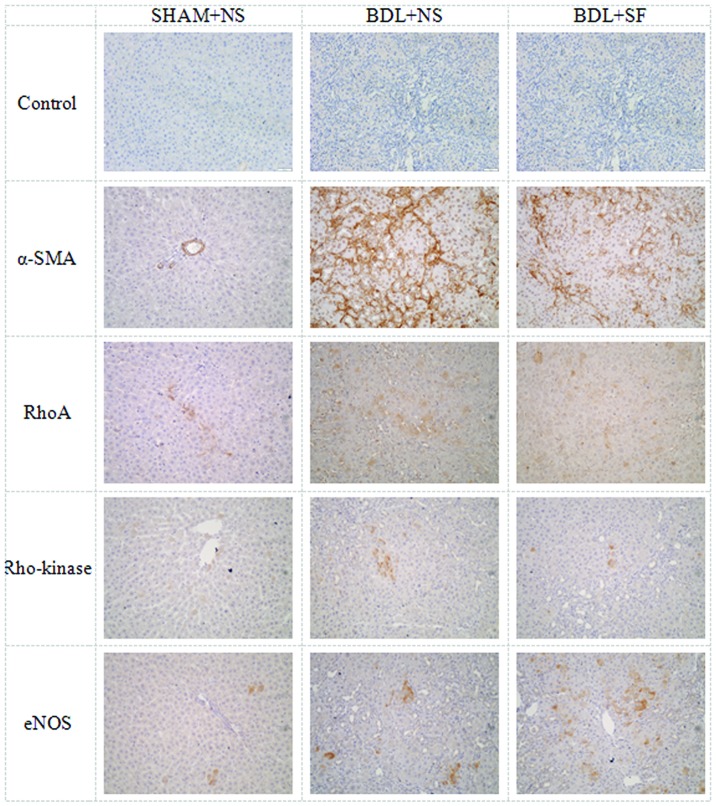
Representative immunohistochemical staining for control, α-smooth muscle actin (α-SMA), RhoA, Rho-kinase and endothelial nitric oxide synthase (eNOS) staining in the 3 groups. Control, α-SMA, RhoA, Rho-kinase and eNOS immunohistochemical staining is shown for the sham-operated + normal saline (SHAM + NS), bile duct ligation + normal saline (BDL + NS) and bile duct ligation + sodium ferulate (BDL + SF) groups.

**Figure 4:**
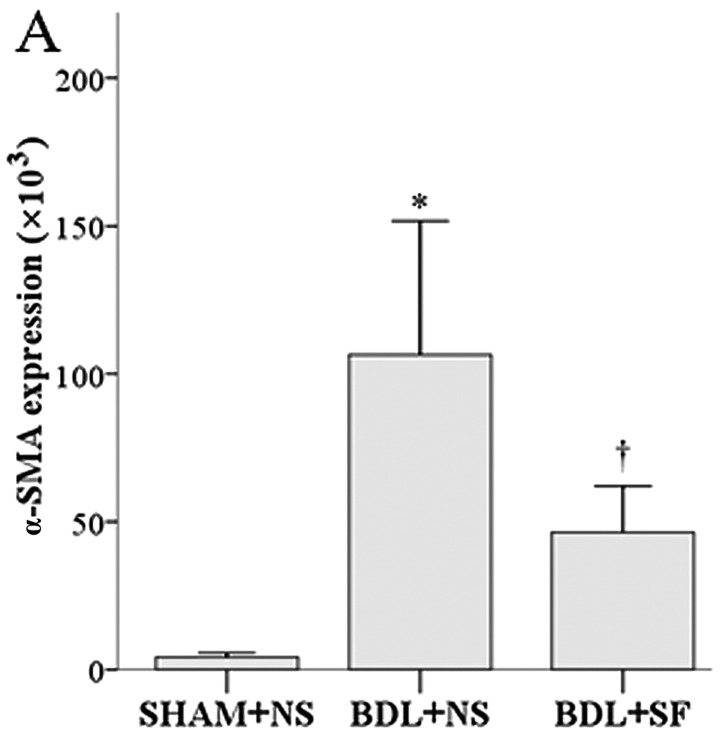

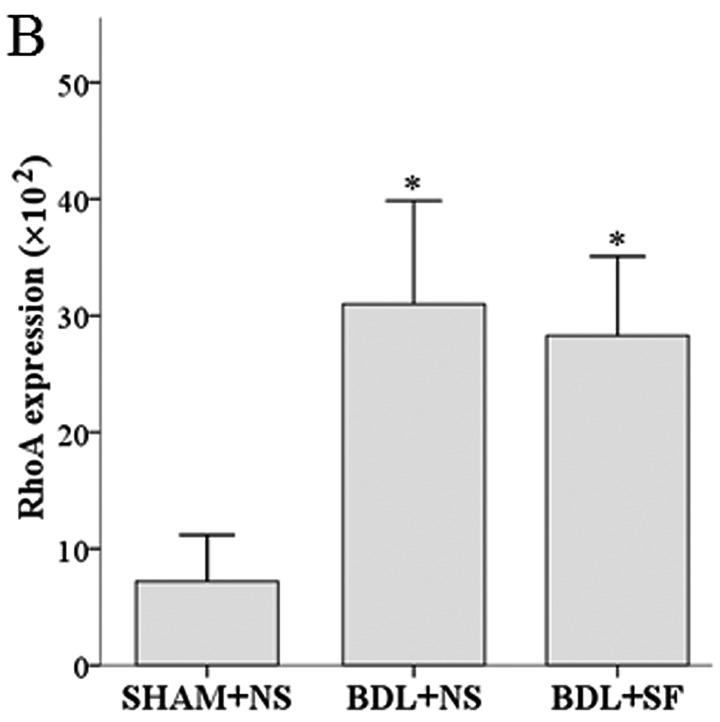

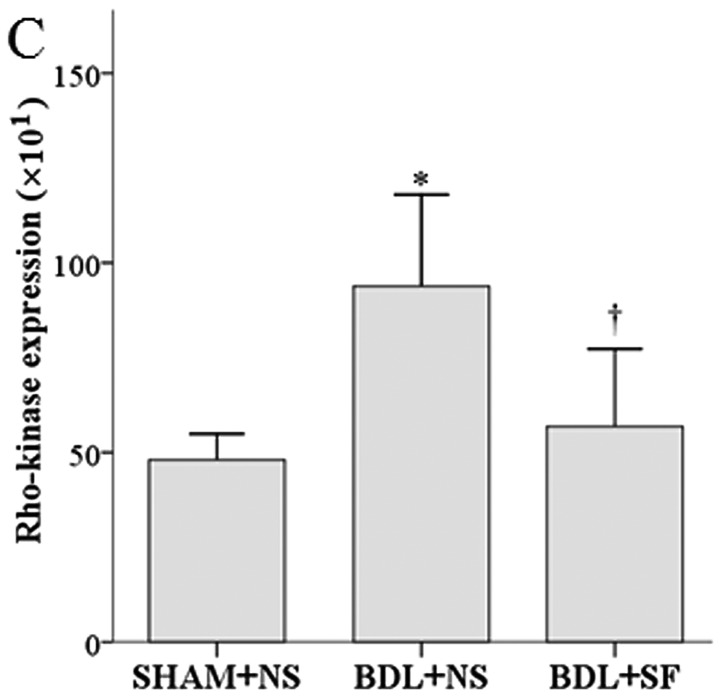

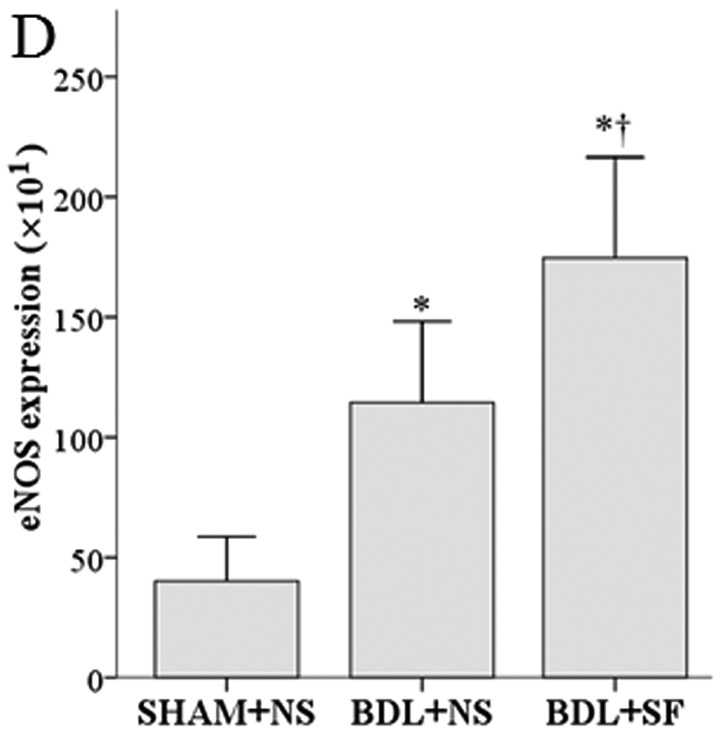
Semi-quantitative analysis of immunohistochemical staining for hepatic α-smooth muscle actin (α-SMA) RhoA, Rho-kinase and endothelial nitric oxide synthase (eNOS) expression in the 3 groups. (A) α-SMA expression. (B) RhoA expression. (C) Hepatic expression of Rho-kinase. (D) Hepatic expression of eNOS, data are expressed as the means ± standard deviation (SD) (n=6). ^*^P<0.05 vs. sham-operated + normal saline (SHAM + NS) group; ^†^P<0.05 vs. bile duct ligation + normal saline (BDL + NS) group.

The histological results for antibody staining for α-SMA, RhoA, Rho-kinase and eNOS in the hepatic cells are shown in Fig. 3, and the corresponding semi-quantitative data are shown in Fig. 4.

SF reduced the high levels of α-SMA and Rho-kinase observed in the cirrhotic rats, but had no significant effect on the high expression of RhoA observed in these animals.

The α-SMA and Rho-kinase expression in the rats in the BDL + NS group was significantly higher than that in the rats in the SHAM + NS and BDL + SF groups (all P≤0.004) (Fig. 4A and C). RhoA expression in the BDL + NS and BDL + SF groups was significantly higher than that in the SHAM + NS group (both P<0.001) (Fig. 4B).

eNOS expression was increased in the cirrhotic rats, and treatment with SF increased eNOS expression even further. eNOS expression in the BDL + NS and BDL + SF groups was significantly higher than that in the SHAM + NS group (both P≤0.001), and that of the BDL + SF group was significantly higher than that of the BDL + NS group (P=0.006) (Fig. 4D).

### Effect of SF/GGPP on the apoptosis of primary HSCs and LX-2 cells

**Figure 5:**
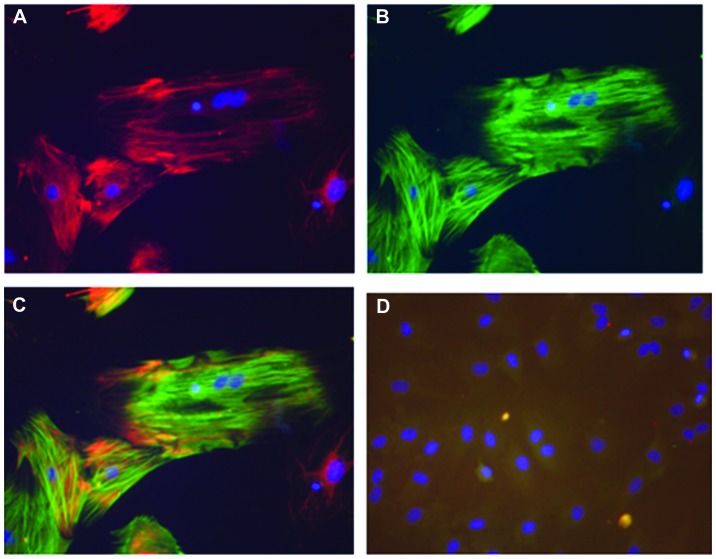
Immunofluorescence images of rat hepatic stellate cells (HSCs) in culture. (A) HSCs stained with DAPI and desmin-specific antibody. Cytoplasm is red and nucleus is blue; (B) HSCs stained with DAPI and α-smooth muscle actin (α-SMA)-specific antibody. Cytoplasm is green and nucleus is blue; (C) HSCs were stained with DAPI, α-SMA and desmin. Cytoplasm shows overlapping red and green fluorescence, nucleus is blue; (D) HSCs stained with DAPI only. Cytoplasm has no fluorescence, nucleus is blue. Magnification, ×200.

**Figure 6:**
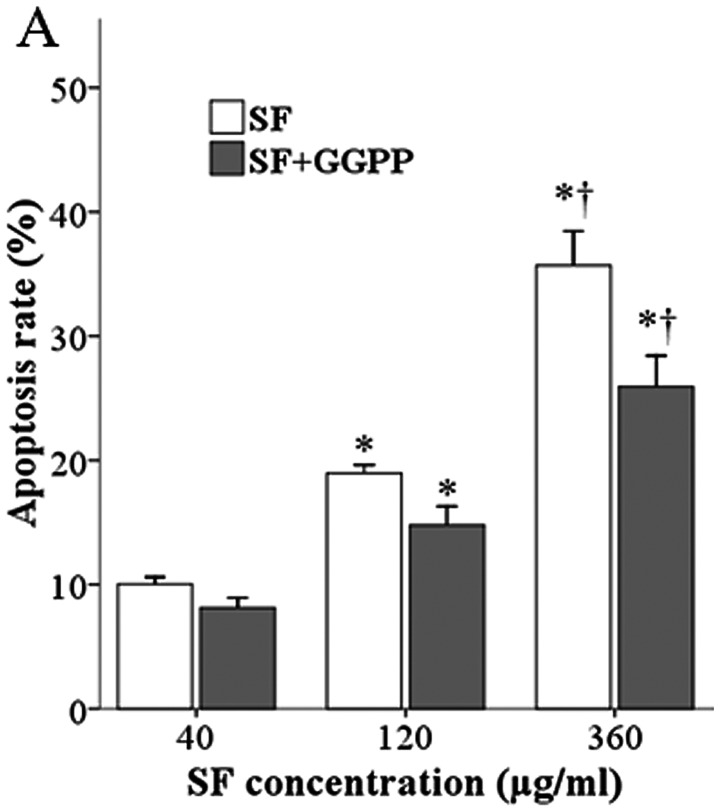

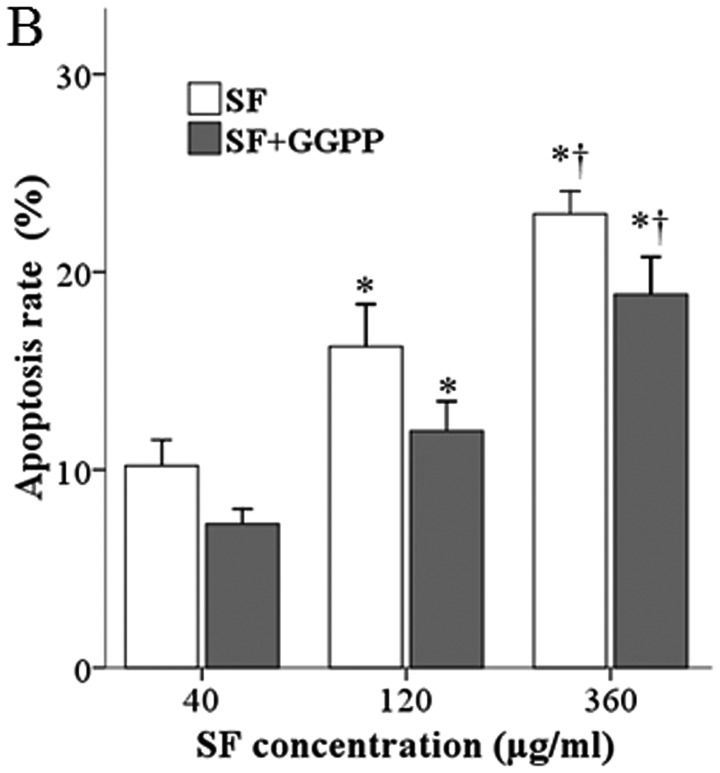
Effects of sodium ferulate (SF) and geranylgeranyl pyrophosphate (GGPP) on the apoptosis of hepatic stellate cells. (A) Rat hepatic stellate cells. (B) LX-2 human hepatic stellate cells. Data are expressed as the means ± standard deviation (SD) (n=3). ^*^P<0.05 vs. SF 40 μg/ml; ^†^P<0.05 vs. SF 120 μg/ml.

Fig. 5 illustrates the DAPI, desmin and α-SMA staining of the rat HSCs in culture, illustrating the activation of these cells. SF produced a concentration-dependent increase in apoptosis in both the rat and human HSCs (Fig. 6) compared to the controls not treated with SF (4.6±0.9 and 4.7±1.4 for rat and human HSCs, respectively; control data not shown). The addition of GGPP, the substance that enables RhoA to translocate from the cytoplasm to the cell membrane and become activated, partly blocked the ferulate-induced increase.

### In situ liver perfusion and the perfusion pressure response to methoxamine hydrochloride

**Figure 7:**
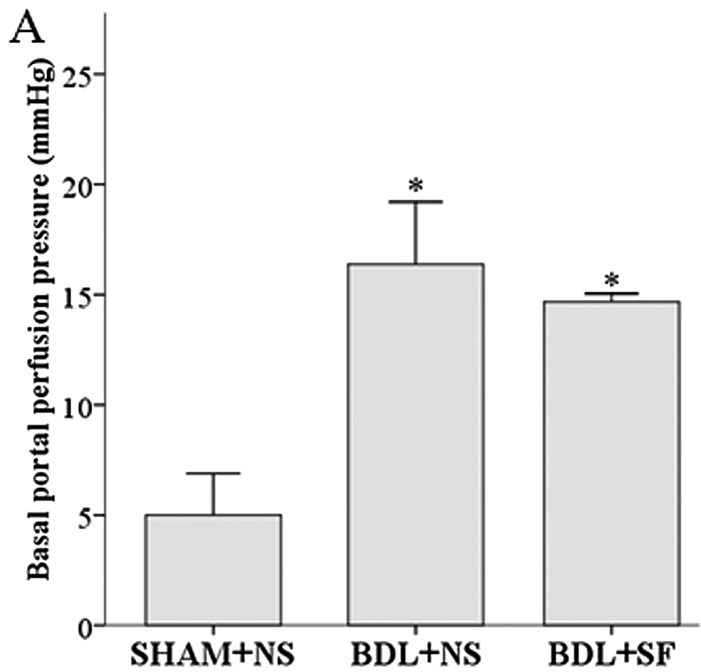

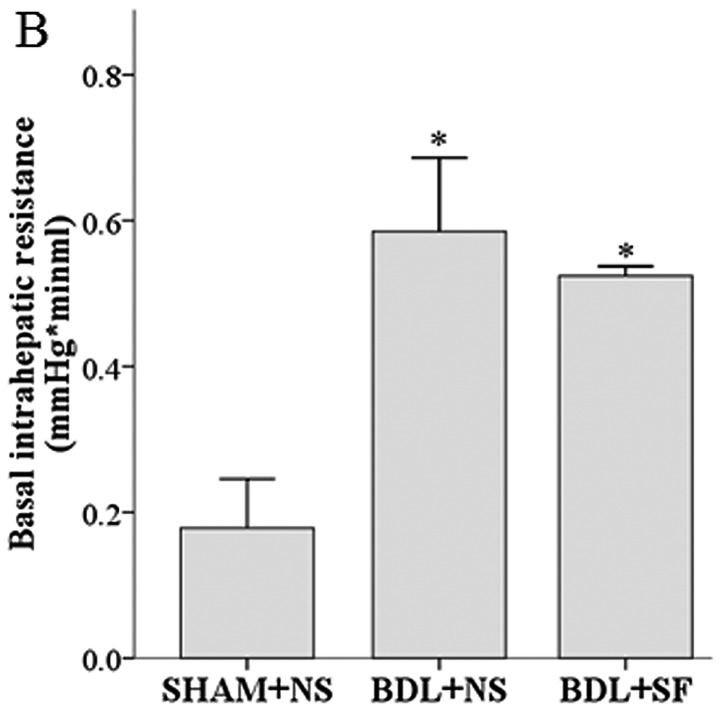
(A) Basal portal perfusion pressure and (B) basal intrahepatic resistance in sham-operated + normal saline (SHAM + NS), bile duct ligation + normal saline (BDL + NS) and bile duct ligation + sodium ferulate (BDL + SF) groups. BDL + NS and BDL + SF groups have significantly higher basal perfusion than the SHAM + NS group. Data are expressed as the means ± standard deviation (SD) (n=10). ^*^P<0.05 vs. SHAM + NS group.

**Figure 8:**
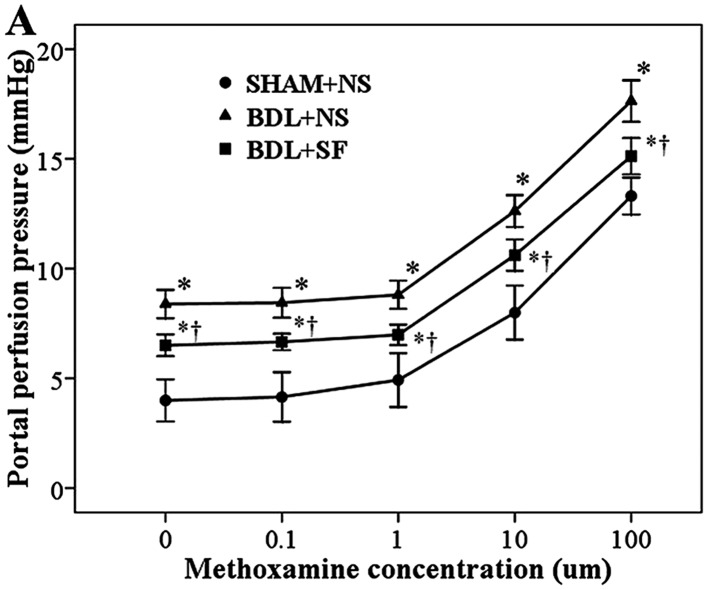

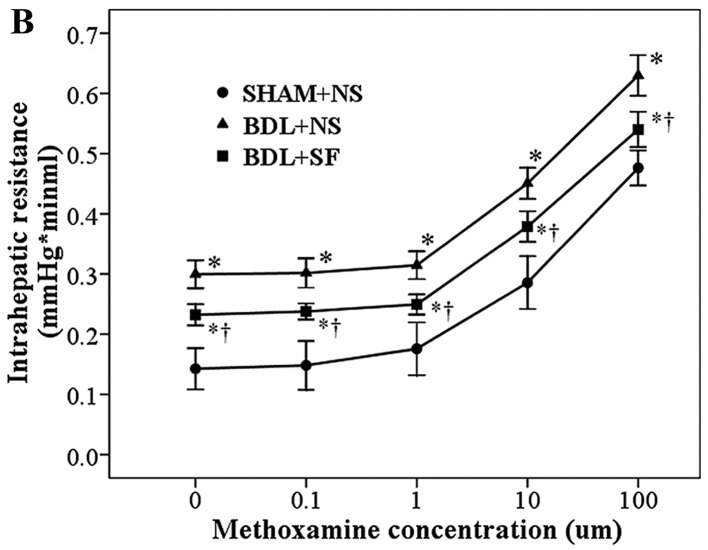
Cumulative concentration-response curves of methoxamine-induced (A) portal perfusion pressure and (B) intrahepatic resistance in sham-operated + normal saline (SHAM + NS), bile duct ligation + normal saline (BDL + NS) and bile duct ligation + sodium ferulate (BDL + SF) groups. SF induced a significant decrease in the response to methoxamine observed in rats subjected to BDL. Data are expressed as the means ± standard deviation (SD) (n=8). ^*^P<0.05 vs. SHAM + NS group at the same methoxamine concentration; ^†^P<0.05 vs. BDL + NS group at the same methoxamine concentration

Basal perfusion pressure and intrahepatic resistance were significantly increased in the cirrhotic rats, and SF had no significant effect on these increases (Fig. 7). SF did, however, significantly decrease the hyperresponsiveness to methoxamine observed in these rats (Fig. 8).

Basal portal perfusion pressure and basal intrahepatic resistance were significantly higher in the BDL + NS and BDL + SF groups compared to the SHAM + NS group (all P<0.001). In all the experimental groups, methoxamine induced a concentration-dependent increase in portal perfusion pressure. Under identical methoxamine concentrations, portal perfusion pressure in the BDL + NS and BDL + SF groups was significantly higher than that of the SHAM + NS group (all P<0.001), and that of the BDL + NS group was significantly higher than that of the BDL + SF group (all P<0.001).

## Discussion

In the present study, we hypothesized that SF, which decreases the synthesis of GGPP, a compound essential for RhoA activation and initiation of the RhoA/Rho-kinase pathway in HSCs, would ameliorate fibrosis and portal hypertension in rats with secondary biliary cirrhosis. SF did decrease fibrosis and the elevated portal pressure, possibly through the inhibition of the Rho-kinase pathway by decreasing Rho-kinase expression. SF also increased the apoptosis of HSCs, and this action may also be mediated through the Rho-kinase pathway, as the addition of the missing initiator of this pathway, GGPP, decreased the ferulate-induced HSC apoptosis.

Portal hypertension, the major factor causing high mortality in cirrhotic patients, is responsible for serious complications such as ascites, esophagogastric varices, hepatorenal syndrome and hepatic encephalopathy (31), and is determined by intrahepatic vascular resistance and portal blood flow (portal pressure = resistance × flow). Activated HSCs, in addition to their role in fibrogenesis, are responsible for the increased vascular resistance obseved in the hepatic sinusoid in cirrhosis, and these actions are thought to be mediated through the RhoA/Rho-kinase pathway. Our results revealed that SF decreased the expression of Rho-kinase, but not that of RhoA in hepatic tissue from cirrhotic rats. RhoA, a member of the GTP-binding protein-Rho GTPase family, exists in both activated GTP-RhoA and stationary GDP-RhoA states, and in the membrane-bound activated state, it activates Rho-kinase. The activation of Rho-kinase exerts two effects that increase portal pressure. One is the inhibition of myosin light chain phosphatase, causing the downstream effect of increasing smooth muscle contraction (9,32,33). The other is the decrease in hepatic eNOS activity, thus increasing the sensitivity of the venules to vasoconstrictors, such as methoxamine. Zhou *et al* (2) previously found that the expression of RhoA and Rho-kinase in the livers of experimental cirrhotic rats was significantly higher than in normal rats. In addition, salvianolic acid, a compound that similar to SF, decreases GGPP synthesis (34), has been reported to lower portal pressure and inhibit HSC contraction by downregulating the RhoA/Rho-kinase pathway (35). Although ferulic acid has previously been reported to decrease blood pressure in hypertensive rats (36) and lower portal pressure in patients with liver cirrhosis (37), the mechanisms responsible for these actions have not yet been elucidated. Therefore, in this study, we examined the effects of SF on the portal pressure of cirrhotic rats by investigating the RhoA/Rho-kinase signaling pathway.

We first examined the effect of SF on the other endpoint of the RhoA/Rho-kinase pathway, fibrosis. We investigated the effects on fibrosis using three methods: i) by comparing fibrous hyperplasia in H&E-stained liver sections, ii) by electron microscopic examination of hepatocyte ultrastructure and the deposition of collagen in the perisinusoidal and intracellular space; and iii) by the semi-quantitative determination of α-SMA. SF markedly decreased both the histological evidence of fibrosis and the elevated α-SMA levels observed in cirrhotic rats. SF had no effect on the serum biochemical indicators of liver fibrosis or on the hepatic hydroxyproline content in bile duct-ligated rats. This discrepancy requires further investigation. In addition, SF promoted HSC apoptosis, and the contribution of decreasing HSC numbers through apoptosis to the decrease in α-SMA and histological evidence of decreased liver injury requires further investigation.

We then assessed the effects of SF on the RhoA/Rho-kinase pathway and found that SF attenuated the increased expression of Rho-kinase observed in cirrhotic rats, but had no effect on the increased expression of RhoA. RhoA must be both membrane-bound and GTP-bound in order to activate Rho-kinase and cause downstream effects. Our assay for RhoA included both GTP-RhoA and GDP-RhoA, and thus could not detect whether or not SF caused a decrease in the activated form of RhoA. GDP-RhoA and GTP-RhoA are two kinds of mutual conversion states; only GTP-RhoA, which transfers to the cell membrane can activate Rho-kinase and cause downstream effects. However, the decrease in the expression of hepatic Rho-kinase in response to SF was an indication that the downstream effects caused by GTP-RhoA were inhibited.

We did not measure the effect of SF on myosin light chain phosphatase, but we did measure its effect on eNOS activity, the other downstream target of the RhoA/Rho-kinase pathway involved in regulating portal pressure. eNOS is broadly distributed and found in myocardial cells, endothelial cells, mast cells and blood cells. eNOS activity causes vascular smooth muscle relaxation and plays a key role in maintaining the steady state of the vascular wall (38). In this study, eNOS activity was increased in the cirrhotic rats, and SF increased the expression of hepatic eNOS even further. Therefore, SF possibly has a positive effect on intrahepatic vascular relaxation through an increase in eNOS expression (9,19,39) and this may be mediated through blocking Rho-kinase activation, for Rho-kinase decreases eNOS and its activated, phosphorylated form (10,19). However, the explanation may not be so simple, as the regulation of eNOS in HSC is complex. Rho-kinase signaling is elevated in HSCs in cirrhosis, but part of the activation is caused by the release of ET-1 from the spleen (40). The farnesoid receptor, a bile-acid-responsive transcription factor, decreases eNOS activity in cirrhosis, but does so through the Rho-kinase receptor in thioacetamide-induced cirrhosis, but not BDL-induced cirrhosis. In BDL-induced cirrhosis, it acts, instead, through DDAH-2, an enzyme responsible for the degradation of asymmetrical dimethylarginine (ADMA), an endogenous inhibitor of eNOS (41). The increase in eNOS in the rats subjected to BDL in this study is inexplicable at this time, as a decrease in hepatic eNOS activity in cirrhosis is considered to be one of the mechanisms responsible for the vascular hyperreactivity to vasoconstrictors observed under this condition.

To examine the effects of SF on intrahepatic vascular resistance, we performed *in situ* perfusion to record the perfusion pressure. SF did not significantly decrease the high basal perfusion pressure observed in cirrhotic rats. However, it partly decreased the hyperresponsiveness to methoxamine observed in cirrhotic livers, possibly through its inhibition of the RhoA/Rho-kinase pathway. The results supported our hypothesis: SF decreased fibrosis and portal hypertension possibly through the inhibition of the Rho-kinase pathway. Further studies are required to explore the role of eNOS and HSC apoptosis in portal pressure.

The parent compound, ferulic acid, has been used to treat a number of clinical conditions (38), but has rarely been used in liver diseases. Ferulic acid inhibits renal tubulointerstitial fibrosis in rats (42), protects against liver injury in mice (10,43–46), downregulates blood pressure in hypertensive rats (36,47) and decreases portal pressure in cirrhotic patients with portal hypertension (37). These studies, and our data showing that SF decreases fibrosis and portal hypertension, suggest that this compound may have potential for use in the treatment of liver disease.

Statins, through their inhibition of cholesterol synthesis, block the same pathway as ferulic acid, are protective against hepatic fibrosis and have been used in clinical practice (48). Ferulic acid is a Chinese herb, and its pharmacological activities have been extensively investigated (49). It has advantages over statins as it also exerts anti-inflammatory and antioxant effects. In addition, statins have side-effects, such as gastrointestinal discomfort, and, as they cause an increase in aminotransferase levels, are not suitable for the treatment of active liver disease (50).

In conclusion, we demonstrate that SF inhibits the hepatic RhoA/Rho-kinase signaling pathway, thus decreasing the activation of HSCs. It also increases eNOS synthesis, ultimately causing hepatic portal pressure decrease in cirrhotic rats. It decreases fibrosis and increases the apoptosis of HSCs. To date, the expression of RhoA, Rho-kinase and eNOS has been studied using a semiquantitative immunohistochemical method. Experiments on these substances at the protein and RNA levels are required. Our preliminary research indicates that ferulic acid may be an effective novel therapeutic agent for the treatment of patients with hepatic cirrhosis with portal hypertension.

## Figures and Tables

**Table I:** General characteristics and serum biochemical parameters of the different experimental groups.

	SHAM + NS	BDL + NS	BDL + SF	P-value
General characteristics	(n=15)	(n=15)	(n=15)	
Body weight (g)	284.3±29.4	237.0±32.6^a^	240.7±33.0^a^	<0.001^b^
Liver weight (g)	11.8±1.4	17.4±3.0^a^	17.0±3.5^a^	<0.001^b^
Spleen weight (g)	0.9±0.2	1.9±0.6^a^	1.7±0.6^a^	<0.001^b^
Liver function	(n=15)	(n=10)	(n=10)	
ALT (U/l)	35.7±13.7	96.5±42.2^a^	89.7±20.3^a^	<0.001^b^
AST (U/l)	115.5±54.3	364.6±231.2^a^	344.8±106.3^a^	<0.001^b^
ALB (g/l)	38.7±3.6	25.2±3.8^a^	26.6±2.8^a^	<0.001^b^
TBIL (μmol/l)	0.5±0.2	113.9±22.3^a^	107.9±50.4^a^	<0.001^b^
DBIL (μmol/l)	0.5±0.7	81.5±40.3^a^	62.4±47.1^a^	<0.001^b^
γ-GT (U/l)	1.8±1.0	82.8±30.1^a^	81.4±38.1^a^	<0.001^b^
Fibrogenesis	(n=15)	(n=10)	(n=10)	
HA (ng/ml)	110.4±18.2	530.0±57.2^a^	512.9±64.7^a^	<0.001^b^
LN (ng/ml)	65.3±2.5	76.1±2.5^a^	76.6±1.6^a^	<0.001^b^
IV-C (ng/ml)	37.3±3.2	45.3±2.3^a^	44.5±1.7^a^	<0.001^b^
PCIII (ng/ml)	34.6±1.4	45.0±1.8^a^	44.5±2.8^a^	<0.001^b^

a P<0.05 indicates a significant difference when compared with the SHAM + NS group;

b P<0.05 indicates a significant difference among the 3 experimental groups.

SHAM, sham-operated; NS, normal saline; SF, sodium ferulate; BDL, bile duct ligation; ALT, alanine aminotransferase; AST, aspartate aminotransferase; ALB, albumin; TBIL, total bilirubin; DBIL, direct bilirubin; HA, hyaluronic acid; LN, laminin; IV-C, collagen type IV; PCIII, procollagen type II peptide. Data are expressed as the means ± standard deviation, and analyzed by one-way ANOVA.

## References

[1] Hennenberg M, Biecker E, Trebicka J (2006). Defective RhoA/Rho-kinase signaling contributes to vascular hypocontractility and vasodilation in cirrhotic rats. Gastroenterology.

[2] Zhou Q, Hennenberg M, Trebicka J (2006). Intrahepatic upregulation of RhoA and Rho-kinase signalling contributes to increased hepatic vascular resistance in rats with secondary biliary cirrhosis. Gut.

[3] Bari K, Garcia-Tsao G (2012). Treatment of portal hypertension. World J Gastroenterol.

[4] Miñano C, Garcia-Tsao G (2010). Clinical pharmacology of portal hypertension. Gastroenterol Clin North Am.

[5] van Beuge MM, Prakash J, Lacombe M (2011). Reduction of fibrogenesis by selective delivery of a Rho kinase inhibitor to hepatic stellate cells in mice. J Pharmacol Exp Ther.

[6] Ikeda H, Nagashima K, Yanase M (2003). Involvement of Rho/Rho kinase pathway in regulation of apoptosis in rat hepatic stellate cells. Am J Physiol Gastrointest Liver Physiol.

[7] Sohail MA, Hashmi AZ, Hakim W (2009). Adenosine induces loss of actin stress fibers and inhibits contraction in hepatic stellate cells via Rho inhibition. Hepatology.

[8] van Beuge MM, Prakash J, Lacombe M, Post E, Reker-Smit C, Beljaars L, Poelstra K (2011). Increased liver uptake and reduced hepatic stellate cell activation with a cell-specific conjugate of the Rho-kinase inhibitor Y27632. Pharm Res.

[9] Trebicka J, Hennenberg M, Laleman W (2007). Atorvastatin lowers portal pressure in cirrhotic rats by inhibition of RhoA/Rho-kinase and activation of endothelial nitric oxide synthase. Hepatology.

[10] Anegawa G, Kawanaka H, Yoshida D (2008). Defective endothelial nitric oxide synthase signaling is mediated by rho-kinase activation in rats with secondary biliary cirrhosis. Hepatology.

[11] Brandão DF, Ramalho LN, Ramalho FS, Zucoloto S, de Martinelli AL, de Silva OC (2006). Liver cirrhosis and hepatic stellate cells. Acta Cir Bras.

[12] Chakraborty JB, Oakley F, Walsh MJ (2012). Mechanisms and biomarkers of apoptosis in liver disease and fibrosis. Int J Hepatol.

[13] Cichoz-Lach H, Celinski K, Slomka M, Kasztelan-Szczerbinska B (2008). Pathophysiology of portal hypertension. J Physiol Pharmacol.

[14] Friedman SL (2008). Mechanisms of hepatic fibrogenesis. Gastroenterology.

[15] Charlton-Menys V, Durrington PN (2008). Human cholesterol metabolism and therapeutic molecules. Exp Physiol.

[16] Lee MH, Cho YS, Han YM (2007). Simvastatin suppresses self-renewal of mouse embryonic stem cells by inhibiting RhoA geranylgeranylation. Stem Cells.

[17] Schmidmaier R, Baumann P, Simsek M, Dayyani F, Emmerich B, Meinhardt G (2004). The HMG-CoA reductase inhibitor simvastatin overcomes cell adhesion-mediated drug resistance in multiple myeloma by geranylgeranylation of Rho protein and activation of Rho kinase. Blood.

[18] Martinez-Sales V, Vila V, Ferrando M, Reganon E (2011). Atorvastatin neutralises the thrombin-induced tissue factor expresion in endothelial cells via geranylgeranyl pyrophosphate. Cytotechnology.

[19] Luo W, Meng Y, Ji HL (2012). Spironolactone lowers portal hypertension by inhibiting liver fibrosis, ROCK-2 activity and activating NO/PKG pathway in the bile-duct-ligated rat. PLoS One.

[20] Piao RL, Brigstock DR, Zhu J, Zhang ML, Gao RP (2012). Clinical significance of connective tissue growth factor in hepatitis B virus-induced hepatic fibrosis. World J Gastroenterol.

[21] Xu Q, Liu X, Chen W, Zhang Z (2010). Inhibiting adenoid cystic carcinoma cells growth and metastasis by blocking the expression of ADAM 10 using RNA interference. J Transl Med.

[22] Jiroutová A, Majdiaková L, Cermáková M, Köhlerová R, Kanta J (2005). Expression of cytoskeletal proteins in hepatic stellate cells isolated from normal and cirrhotic rat liver. Acta Medica (Hradec Kralove).

[23] Kim KY, Choi I, Kim SS (2000). Purification and characterization of a novel inhibitor of the proliferation of hepatic stellate cells. J Biochem.

[24] Olaso E, Arteta B, Benedicto A, Crende O, Friedman SL (2011). Loss of discoidin domain receptor 2 promotes hepatic fibrosis after chronic carbon tetrachloride through altered paracrine interactions between hepatic stellate cells and liver-associated macrophages. Am J Pathol.

[25] March S, Graupera M, Rosa Sarrias M, Lozano F, Pizcueta P, Bosch J, Engel P (2007). Identification and functional characterization of the hepatic stellate cell CD38 cell surface molecule. Am J Pathol.

[26] Vairetti M, Richelmi P, Bertè F, Currin RT, Lemasters JJ, Imberti R (2006). Role of pH in protection by low sodium against hypoxic injury in isolated perfused rat livers. J Hepatol.

[27] Kukan M, Szatmáry Z, Lutterová M, Kuba D, Vajdová K, Horecký J (2004). Effects of sizofiran on endotoxin-enhanced cold ischemia-reperfusion injury of the rat liver. Physiol Res.

[28] Vairetti M, Ferrigno A, Carlucci F (2009). Subnormothermic machine perfusion protects steatotic livers against preservation injury: a potential for donor pool increase?. Liver Transpl.

[29] Kim MY, Baik SK, Lee SS (2010). Hemodynamic alterations in cirrhosis and portal hypertension. Korean J Hepatol.

[30] Reynaert H, Urbain D, Geerts A (2008). Regulation of sinusoidal perfusion in portal hypertension. Anat Rec (Hoboken).

[31] Al-Busafi SA, McNabb-Baltar J, Farag A, Hilzenrat N (2012). Clinical manifestations of portal hypertension. Int J Hepatol.

[32] Bishop AL, Hall A (2000). Rho GTPases and their effector proteins. Biochem J.

[33] Wang Y, Zheng XR, Riddick N, Bryden M, Baur W, Zhang X, Surks HK (2009). ROCK isoform regulation of myosin phosphatase and contractility in vascular smooth muscle cells. Circ Res.

[34] Ho JH, Hong CY (2011). Salvianolic acids: small compounds with multiple mechanisms for cardiovascular protection. J Biomed Sci.

[35] Xu H, Zhou Y, Lu C, Ping J, Xu LM (2012). Salvianolic acid B lowers portal pressure in cirrhotic rats and attenuates contraction of rat hepatic stellate cells by inhibiting RhoA signaling pathway. Lab Invest.

[36] Ardiansyah, Ohsaki Y, Shirakawa H, Koseki T, Komai M (2008). Novel effects of a single administration of ferulic acid on the regulation of blood pressure and the hepatic lipid metabolic profile in stroke-prone spontaneously hypertensive rats. J Agric Food Chem.

[37] Huang Z, Wei W, Zhong Q (2008). Effect of sodium ferulate on hemodynamics in hepatic cirrhosis patients with portal hypertension. Zhongguo Zhong Xi Yi Jie He Za Zhi.

[38] Dudzinski DM, Michel T (2007). Life history of eNOS: partners and pathways. Cardiovasc Res.

[39] Shiga N, Hirano K, Hirano M, Nishimura J, Nawata H, Kanaide H (2005). Long-term inhibition of RhoA attenuates vascular contractility by enhancing endothelial NO production in an intact rabbit mesenteric artery. Circ Res.

[40] Uehara H, Akahoshi T, Kawanaka H (2012). Endothelin-1 derived from spleen-activated Rho-kinase pathway in rats with secondary biliary cirrhosis. Hepatol Res.

[41] Verbeke L, Farre R, Trebicka J (2013). Obeticholic acid, a farnesoid-X receptor agonist, improves portal hypertension by two distinct pathways in cirrhotic rats. Hepatology.

[42] Meng LQ, Tang JW, Wang Y (2011). Astragaloside IV synergizes with ferulic acid to inhibit renal tubulointerstitial fibrosis in rats with obstructive nephropathy. Br J Pharmacol.

[43] Alam MA, Sernia C, Brown L (2013). Ferulic acid improves cardiovascular and kidney structure and function in hypertensive rats. J Cardiovasc Pharmacol.

[44] Kim HY, Park J, Lee KH, Lee DU, Kwak JH, Kim YS, Lee SM (2011). Ferulic acid protects against carbon tetrachloride-induced liver injury in mice. Toxicology.

[45] Rukkumani R, Aruna K, Suresh Varma P, Padmanabhan Menon V (2004). Hepatoprotective role of ferulic acid: a dose-dependent study. J Med Food.

[46] Xu X, Xiao H, Zhao J, Zhao T (2012). Cardioprotective effect of sodium ferulate in diabetic rats. Int J Med Sci.

[47] Wang BH, Ou-Yang JP (2005). Pharmacological actions of sodium ferulate in cardiovascular system. Cardiovasc Drug Rev.

[48] Lowyck I, Fevery J (2007). Statins in hepatobiliary diseases: effects, indications and risks. Acta Gastroenterol Belg.

[49] Lin Z, Gu J, Xiu J, Mi T, Dong J, Tiwari JK (2012). Traditional chinese medicine for senile dementia. Evid Based Complement Alternat Med.

[50] Holmberg B, Brännström M, Bucht B, Crougneau V, Dimeny E, Ekspong A, Granroth B, Gröntoft KC, Hadimeri H, Ingman B, Isaksson B, Johansson G, Lindberger K, Lundberg L, Mikaelsson L, Olausson E, Persson B, Welin D, Wikdahl AM, Stegmayr BG (2005). Safety and efficacy of atorvastatin in patients with severe renal dysfunction. Scand J Urol Nephrol.

